# How does embedded implementation research work? Examining core features through qualitative case studies in Latin America and the Caribbean

**DOI:** 10.1093/heapol/czaa126

**Published:** 2020-11-06

**Authors:** N Ilona Varallyay, Sara C Bennett, Caitlin Kennedy, Abdul Ghaffar, David H Peters

**Affiliations:** 1 Department of International Health, Johns Hopkins School of Public Health, 615 N Wolfe St, Baltimore, MD 21205, United States; 2 Social and Behavioral Interventions Program, Department of International Health, Johns Hopkins School of Public Health, 615 N Wolfe St, Baltimore, MD 21205, United States; 3 The Alliance for Health Policy and Systems Research at the World Health Organization, 20 avenue Appia, 1211 Geneva, Switzerland

**Keywords:** Implementation research, embedded research, decision-maker-led research, evidence coproduction, research–practice partnerships, collaborative research partnerships, evidence-informed decision-making, evidence-to-action, knowledge translation, health policy and systems research, low- and middle-income countries, Latin America and the Caribbean

## Abstract

Innovative strategies are needed to improve the delivery of evidence-informed health interventions. Embedded implementation research (EIR) seeks to enhance the generation and use of evidence for programme improvement through four core features: (1) central involvement of programme/policy decision-makers in the research cycle; (2) collaborative research partnerships; (3) positioning research within programme processes and (4) research focused on implementation. This paper examines how these features influence evidence-to-action processes and explores how they are operationalized, their effects and supporting conditions needed. We used a qualitative, comparative case study approach, drawing on document analysis and semi-structured interviews across multiple informant groups, to examine three EIR projects in Bolivia, Colombia and the Dominican Republic. Our findings are presented according to the four core EIR features. The central involvement of decision-makers in EIR was enhanced by decision-maker authority over the programme studied, professional networks and critical reflection. Strong research–practice partnerships were facilitated by commitment, a clear and shared purpose and representation of diverse perspectives. Evidence around positioning research within programme processes was less conclusive; however, as all three cases made significant advances in research use and programme improvement, this feature of EIR may be less critical than others, depending on specific circumstances. Finally, a research focus on implementation demanded proactive engagement by decision-makers in conceptualizing the research and identifying opportunities for direct action by decision-makers. As the EIR approach is a novel approach in these low-resource settings, key supports are needed to build capacity of health sector stakeholders and create an enabling environment through system-level strategies. Key implications for such supports include: promoting EIR and creating incentives for decision-makers to engage in it, establishing structures or mechanisms to facilitate decision-maker involvement, allocating funds for EIR, and developing guidance for EIR practitioners.

**KEY MESSAGES**

- Our study reveals the potential impact of decision-maker-led research partnerships on research use and programme improvement, when well-supported.
- Our findings highlight the importance of the central involvement of decision-makers in EIR; decision-maker authority over the programme studied, professional networks and critical reflection underpin this role.
- Critical enabling factors of the research–practice partnerships relate to commitment, clear and shared purpose and representation of diverse perspectives.
- A research focus on implementation is fundamental to the EIR approach, demands proactive engagement and critical reflection by decision-maker principal investigators in conceptualizing the research, and can lead to identification of opportunities for direct evidence-informed action by decision-makers.

## Introduction

Around the world, there is growing importance placed on improving the delivery of evidence-informed interventions to achieve population health outcomes. This often-cited narrowing of the ‘know-do’ gap is particularly relevant for low-income settings where careful allocation of scare resources is needed to maximize health benefits and achieve the UN Sustainable Development Goals (Pantoja *et al*., 2018). Implementation research (IR) is gaining recognition (Theobald *et al*., 2018) as a potentially impactful way to address this gap by generating locally relevant evidence to inform feasible, effective implementation strategies. While IR has multiple definitions (Odeny *et al*., 2015), there is broad consensus that it is an applied approach to scientific inquiry concerned primarily with the ‘how’ and ‘why’ of implementation (Peters *et al*., 2013). IR seeks to strengthen the delivery of programmes, policies and practices in routine settings, addressing issues around effectiveness, efficiency, quality, equity and sustainability of implementation, to ultimately improve population health (Panisset *et al*., 2012).

One approach to IR that has shown promise is the embedding of IR in the ‘real world’ of implementation (Ghaffar *et al*., 2017; Langlois *et al*., 2017; Tran *et al*., 2017). However, the notion of ‘embedding’ has taken on different meanings (Olivier *et al*., 2017): embedding individual researchers within health service delivery settings (Vindrola-Padros *et al*, 2017; Wolfenden *et al*., 2017; Cheetham *et al*., 2018); embedding research at the organizational level (Koon *et al*., 2013); embedding (institutionalizing) research into programme/policy processes and/or budgets ( Olivier *et al*., 2017 ). Less common are approaches that seek to embed research *within* practice, through decision-maker-led research partnerships, whereby the knowledge ‘user’ (i.e. decision-makers, managers, implementers) is also knowledge ‘producer’. Founded on principles of collaborative research–practice partnerships ( Kogan and Henkel, 1983 cited in Denis and Lomas, 2003; Hanney *et al*., 2003; Ross *et al*., 2003) and coproduction theory (Heaton *et al*., 2015; Vindrola-Padros *et al*, 2017; Beckett *et al*., 2018 ), engaging the ‘knowledge user’ in research has been shown to positively influence the impact of the evidence produced (Kok *et al*., 2016 ; Williamson *et al*., 2019). In this paper, we conceptualize embedded IR as health system decision-makers taking a prominent role throughout various stages of the research process—starting from the identification of the need for research and the specific implementation problem, to the framing of research questions, data collection and interpretation of findings (Ghaffar *et al*., 2017; Tran *et al*., 2017). Equally critical is the role of these key actors in stimulating the use of the evidence in their policy and programmatic decisions.

The Improving Programs through Embedded Implementation Research (iPIER) programme was established to promote decision-maker-led IR partnerships in Latin America and the Caribbean (LAC). The iPIER model of embedded implementation research (EIR) highlights several core features (see Box 1): health system decision-makers leading research as principal investigators (PIs), formation of collaborative research partnerships, positioning research within programmes processes and a research focus on implementation issues (Tran *et al*., 2017; Varallyay *et al*., 2020) . These features are expected to ensure relevance of the research for programme improvement and facilitate the use of evidence for programme improvement. To date, the iPIER initiative has generated multiple lessons about embedded IR endeavours, including early indications of its potential to promote evidence-informed programme decision-making (PAHO, 2017; Langlois *et al*., 2019). However, the knowledge base about how such embedded IR approaches work is still nascent.

**Box 1 EIR features**

**Role of Decision-makers**

EIR in the iPIER programme places health system implementers—policy-makers, district health officers, programme managers, front-line health workers (decision-makers)—in a central role within the research, providing leadership and direction throughout the endeavour. DMs are therefore both producers and consumers of the research.

**Collaborative research partnership**

EIR requires strong partnership, ideally between health system decision-makers and researchers, throughout the research and post-research stages. This is understood as a form of ‘interaction’ that may benefit evidence-informed decision-making.

**Positioning of research within programme**

For iPIER, the research should focus on existing programmes, integrating the research and programme processes such that their respective activities and cycles are aligned and research is ‘conducted as a part of the implementation process’ (iPIER) or within the context of application of findings.

**Implementation focus**

Research questions address issues related to the delivery of programmes or policies (i.e. how related activities and processes are carried out in different contexts, considering wider health system factors that affect their delivery) and are responsive to the information needs of programme/policy decision-makers.

The WHO Alliance for Health Policy and Systems Research (Alliance) supported an external evaluation of the iPIER initiative, the larger study on which this paper is based, to assess whether and how EIR initiatives such as iPIER can catalyse processes to promote the use of research evidence for programme/policy implementation improvements. While core features of embedded IR have been proposed through this initiative, these have not been examined systematically to understand the importance of each feature in the overall evidence-to-action endeavour. Such evidence is important not only for the design and implementation of similar embedded approaches, but also for determining when such approaches are feasible and warranted. The purpose of this paper is to study whether and how key EIR features (Box 1) influence the implementation and outcomes of the research conducted in three distinct settings. Specifically, we examine: (1) whether and how EIR features are put into practice by the research partnerships, (2) how putting these features into practice affects the experience of participants in each case and (3) the learning these cases offer about key enabling conditions for EIR.

## Methods

### Research context

The most recent round of iPIER was implemented from September 2016 to September 2017 through a joint initiative of the Pan American Health Organization and the Alliance. In this period seven small grants (research projects of US$30–35 000) were disbursed across seven LAC countries^1^ to support collaborative IR partnerships aimed at improving existing health programmes. As a funding requirement, ‘implementers, such as policy-makers, district health officers, programme managers, and front-line health workers’ (referred to collectively in this paper as decision-makers) had to participate as lead investigators. Unique to this initiative was the explicit expectation that study teams would use the research findings to inform programme improvements, beyond the grant timeframe if necessary. All teams received additional expert technical assistance (TA) throughout the research process from research institutions in LAC.

### Study design

A prospective, qualitative comparative case study design was employed, using a constructivist approach. We developed and applied a conceptual framework on embedded IR (NI Varallyay *et al*., submitted for publication) that outlines the core EIR features, the expected processes and outcomes, contextual factors and various hypothesized underlying constructs for EIR (Supplementary Appendix 1). In this paper, we focus on studying the framework’s four core EIR features (Box 1), which in combination reflect the underlying rationale of EIR and are expected to contribute to successful EIR.

### Case selection

A ‘case’ was defined as a collaborative research project that involves health system decision-makers as investigators and conducts IR with the explicit aim of improving a real-world health programme or policy. Cases were selected purposively from the seven 2016 to 2017 iPIER projects (Supplementary Appendix 2) to capture one of two contrasting scenarios: (1) ‘most likely’ cases exhibiting key features postulated in the theory and therefore expected to succeed, and (2) ‘least likely’ cases lacking one or more of the fundamental EIR features, and therefore expected to diverge from the theorized pathway (Odell, 2001; Flyvbjerg, 2006). This approach was driven by the overall goal of the larger evaluation towards theory building, examining key assumptions in the EIR conceptual framework (Gilson, 2012; Yin, 2014).

Based on characteristics of the cases^2^ that were expected to influence the creation, uptake and application of evidence, Colombia and the Dominican Republic (DR) were selected as ‘most likely’ cases and Bolivia was selected as a ‘least likely’ case (see Table 1). 

**Table 1:** Summary of research projects for each case

	**Bolivia**	**Colombia**	**DR**
	Expected ‘least likely’ case	Expected ‘most likely’ case	Expected ‘most likely’ case
**Targeted programme**	*Chispitas* micronutrient supplement (under the national micronutrient strategy)	PTM cervical cancer programme, focusing on the screening component	National FPP within the MoPH Sexual and Reproductive Health Program, focusing on male contraception component
**Health system level**	Sub-municipal level study	Municipal level study	National level study
**Research team affiliations— decision-maker**	**PI**: Frontline health worker: Paediatrician at secondary level municipal hospital **Co-PI 1**: Director of Municipal Health Office at outset; later changed **Co-PI 2**: Technical Coordinator of the health network (study site) at outset; later changed	**PI**: Director of one of five Municipal health networks (highest level authority) **Co-PI**: Senior Technical advisor to the Municipal Health Secretary	**PI**: Coordinator of Office of Gender Equity and Development, Division of Planning (MoPH) **Co-PI 1**: Technical Manager for Sexual and Reproductive Health and Family Planning, Division of Maternal, Child and Adolescent Health (MoPH); Instructor at School of Public Health at Universidad Autonoma of Santo Domingo
**Research team affiliations— research partner^a^**	None	None	**Co-PI 2**: Director of the Institute for Investigation and Study on Gender and Family, Universidad Autonoma of Santo Domingo **Co-PI 3**: Researcher at the Institute for Investigation and Study on Gender and Family, Universidad Autonoma of Santo Domingo [initially]
**Research objective**/**questions**	To understand the barriers and facilitators related to consumption and adherence of the *Chispitas* in children 6–23 months	To identify the strategies related to the access and quality of care in the public health services of Cali that may affect coverage of cervical cancer screening services	To identify mechanisms in the implementation of the FPP that facilitate or constitute barriers to the effective integration of men as a beneficiary population
**Methods**	Qualitative (semi-structured interviews, focus groups and direct observations)	Qualitative (semi-structured interviews, direct observations, focus groups and document review)	Mixed methods (1) Review of the literature (2) Qualitative: semi-structured interviews focus groups (3) Quantitative: survey of health care providers
**Research findings**	- Health worker level: lack of updated knowledge/capacity about *Chispitas* - Beneficiary level: lack of acceptability of *Chispitas* product for practical and cultural/ behavioural reasons - Many of these findings suggest that the ‘intervention product’ (the *Chispitas*) was not successfully piloted for local/cultural acceptability	− Barriers: disconnect between perspective of service providers and service users with regard to access; request among service users for more human-centred care and greater integration with other health programmes; non-users mention cultural beliefs and previous negative experiences with health system as barriers	- The findings confirm what was tacit ly known about the FP programme: there is clear absence of a gender lens within the FP programme (while known anecdotally and through their experience as decision-makers, there was no documented evidence of this gap) - Also revealed interest among men in male contraception [demand]
**Policy**/**programme recommendations**	- No changes to the actual implementation of the intervention, per se; more strongly focused on sensitizing mothers about the importance of the *Chispitas* to reduce anemia and also demonstrations on how they should be used (demand side focused) - Focus on: (1) health worker capacity development (2) carry out demonstrations of *Chispitas* preparation for mothers; on the whole, recommendations are very broad, not clearly actionable	- Focus on health work force capacity to improve service quality; strategizing among the administrators and managers to improve coverage; health information system strengthening and improved monitoring/analysis of relevant indicators; need for additional research on quality of services to be organized by ESE managers - Developed by iPIER research team, without external consultation	- Recommendations span across a wide range of strategies to communicate/educate about, build capacity for service delivery, create strategic alliances and establish norms for male contraception - These are largely drawn from the responses of the decision-makers and health professionals in study interviews - Additional recommendations emerged during the action planning workshop post-dissemination meeting

a Partners were considered ‘researchers’ if their primary professional role was formally affiliated with academic or other research institutions.

### Data collection

Data collection for the case studies was guided by the conceptual framework (Supplementary Appendix 1) and included semi-structured key informant interviews, document review, researcher memos and analysis of secondary qualitative data (see Table 2). The lead author collected, coded and analysed all data in an iterative manner, marked by three rounds of interviews starting in August 2018; the final round of interviews was completed between April and July 2019. The different rounds of data collection enabled the study to track evolutions in EIR processes and impact over time. All interviews were conducted by the lead author in Spanish, audio recorded with consent, transcribed and complemented by memos taken during and after interviews to capture analytical and methodological reflections throughout the research. Interview guides were developed based on the embedded IR conceptual framework, piloted and adapted for each respondent category and stage of the process (see Supplementary Appendix 3). A total of 51 initial and follow-up interviews were conducted with 37 respondents, including all active co-investigators (remotely by Skype) as well as external system stakeholders (in-person) (Table 2). Interview duration ranged from 35 to 85 min, with most lasting about an hour. Our analyses were complemented by review of project documentation and qualitative data from 28 previously conducted interviews which focused on the iPIER research phase. The nature of this prospective case study design and multiple rounds of interviews allowed us to reach saturation in terms of responding to the research questions about the four EIR features. Ethical approval for this study was obtained from the authors’ institute; each EIR project also received local ethical approval. 

**Table 2:** Summary of interview respondent categories by case

Respondent category	Bolivia	Colombia	DR
Research team			
Decision-maker co-PI	3^a^	2^a^	2^a^
Researcher co-PI	None	None	2^a^
External health system stakeholders			
Public sector (e.g. MOH /government)	7	11	5
Other^b^	n/a	1	4
Total number of respondents	10	14	13
Total number of interviews conducted	16	18	17

a Indicates that at least one follow-up interview was conducted; note that in round three of data collection, it was not possible to interview the two co-investigators on the Bolivia team nor the two researchers on the DR team.

b ‘Other’ includes actors in the health system that are not formally affiliated with the national MOH, such as private health insurance companies, professional associations, non-governmental organizations; composition varied according to context.

### Data management and analysis

Using MAXQDA data management software (version 18.1.1), an iterative, deductive approach to analysis was used, whereby interviews were coded as they were transcribed, according to an *a priori* coding structure based on the conceptual framework constructs. Throughout coding, analytical memos were developed to document initial reflections emerging from the data as well as observations about the analytical process.

We first examined the experience of each case individually, using thematic analysis ( Braun and Clarke, 2006 ) to develop descriptive case profiles. The lead author summarized and charted coded data from the various sources into case study matrices organized around the conceptual framework stages ( Ritchie and Spencer, 1994 ; Gale *et al.*, 2013); this process was repeated at each round of data collection. The framework approach helped to expand on *a priori* analytical concepts and identify more nuanced sub-themes related to study team characteristics, the processes they employed, key effects and contextual factors. Subsequently, we reviewed matrix elements specifically related to the EIR features to draw linkages between their operationalization and the processes and outcomes of each case, identifying key supporting conditions through this process.

We then conducted cross-case analysis to develop theoretically generalizable conclusions about the value and importance of the EIR attributes in advancing evidence-informed decision-making. Comparison of the characteristics and experience of each case helped to distill evidence about key conditions for EIR features and the potential effects of each feature.

## Results

We first present a descriptive overview of each case (supplemented by details about EIR features in text boxes), followed by a comparative cross-case analysis to distill critical dimensions of each EIR feature. 

### Case study descriptions

#### *Case 1: Bolivia* Chispitas *micronutrient supplementation*

In Bolivia, qualitative methods were used to examine the barriers and facilitators related to consumption of and adherence to *Chispitas*—the locally produced micronutrient supplements for prevention of anemia in children 6–23 months, internationally known as ‘Sprinkles’. The research idea originated from a health system paediatrician (PI) who identified the problem, sought out municipal health system decision-makers to participate as co-investigators, and led the overall endeavour. While these co-investigators contributed by facilitating access to key stakeholders, they noted that they were unable to continue participating actively in the research due to being transferred to new regions. The IR study generated evidence that a range of health system stakeholders accepted as reflecting the reality on the ground [‘many of the (departmental health offices) identified with these results’—Central level decision-maker], y*et al*so surprised some frontline health workers who assumed their efforts to promote *Chispitas* had been more effective.

The PI led an intensive, proactive strategy to engage a wide range of stakeholders at various levels of the system, starting from protocol development through dissemination of findings. These efforts had the strongest impact at the local level, where the PI and frontline nutrition health workers reported undertaking numerous small-scale, *ad hoc* actions in response to findings—primarily sensitization of health workers and beneficiaries. PI comments suggest that at central level, the absence of a coordinated strategy to engage key stakeholders in review of the evidence for joint problem-solving hampered a more system-level response. Our findings indicate that this project raised attention to an issue perceived to be critical by key system stakeholders, illuminated previously overlooked root causes of the problem, and stimulated re-activation of a high-level decision-making space (Ministry of Health, MOH Anemia Roundtable). While the PI mentioned having attempted to engage this roundtable during the research, its relatively nascent structure, the irregularity of meetings and its limited acting power appear to have stifled these efforts to promote use of research. The political context complicated this endeavour, most notably in terms of the decentralized, yet politically fragmented governance system, as noted repeatedly by the PI and co-investigators. In terms of actions triggered by the findings, while no respondents mentioned widespread change, frontline health workers in the study area were motivated [‘I am very interested (in the results). I want to take action’—municipal health worker] and ‘got their hands dirty’ (central level decision-maker) in conducting one-off corrective activities, including an informal study on local anemia levels and sensitizations on *Chispitas*, with support from central level (See Box 2 for detailed descriptions of EIR features).

**Box 2 Detailed description of EIR features in Bolivia Case**

**Role of decision-makers**

As a frontline health worker at a first-level municipal hospital, the decision-maker PI was positioned one level removed from that of *Chispitas* implementation (i.e. primary health facility or community level). The decision-maker co-investigators held positions of authority in health system management and coordination at municipal and sub-municipal levels. While they made a valuable contribution at the outset, they assumed a more passive advisory role in responding to needs identified by the PI until they were both transferred. The PI brought an insider perspective, which coupled with her highly respected professional status, allowed her to gain the ear of both local frontline practitioners as well as the MOH nutrition unit. However, given her position within the system hierarchy, the PI remarked that she felt limited in the roles she could assume related to wider coordination and stewardship for research-based problem-solving. She also mentioned a potential tradeoff of this insider status in introducing bias to the research, which led to the decision not to involve DMs (herself included) in data collection in certain instances.

**Collaborative research partnership**

Despite the initial collaborative partnership arrangement, this case was not able to maintain this structure. Following the reassignment of the DMs to new regions, the partnership began to dismantle as the DMs gradually disengaged. The PI did not mention any attempt to restructure the research team, leaving her solely responsible for the endeavour.

**Positioning of research within programme**

At the time of this study, the *Chispitas* intervention was not situated within a formal programme (i.e. a clear organizational structure, leadership hierarchy, established decision-making processes); instead it constituted one of many health strategies at municipal level. While the municipality assumed responsibility for the purchase and distribution of *Chispitas*, implementation was done by frontline providers with what the PI describes as limited support. This appears to have presented a challenge in identifying an appropriate entry point by which to integrate research and programme processes. Furthermore, the apparent weak interest in responding to study findings by the new health network coordinator (replacing the transferred DM PI) based on reported human resources constraints posed challenges.

**Implementation focus**

As initially formulated, the research question focused on exploring barriers to consumption among mothers/ caregivers of children 6–23 months; with TA support, the focus was expanded to include perceptions of service providers and local level managers. The results documented in the final project report highlighted problems not just in implementation (e.g. lack of capacity among health care providers in delivery of *Chispitas*), but also concerning the suitability of the intervention in the local setting (e.g. lack of acceptability of the *Chispitas* product by mothers due to both practical and cultural barriers). Recommendations focused on strategies to sensitize mothers about the use of *Chispitas*, through both building capacity of health workers to deliver appropriate, targeted messages and conducting demonstrations of *Chispitas* preparation for mothers. While local level stakeholders expressed receptivity to study findings, the response at central level was less decisive. Despite clear expressions of support for the study from central level decision-makers, our interviews suggest no concrete actions have been taken to address findings.

#### Case 2: Colombia cervical cancer screening

The Colombia team employed qualitative methods to identify the strategies related to the access and quality of care that affect coverage of cervical cancer screening services in the *Por Ti Mujer* (PTM) programme—a programme implemented within one health network that catered to eligible populations from the entire municipality seeking cervical cancer treatment and care. The PI reported that she conceived of the study, drawing on routine monitoring data to identify problems around coverage of eligible women in the cervical cancer screening programme offered in the health network she directed. While both co-investigators expressed their interest in initiating a partnership to respond to the call for proposals, the PI ultimately assumed primary leadership and remained actively involved throughout research and brief post-research phase with continued support from the co-PI.

Our analysis suggests that this study generated actionable, demand-driven evidence on adjustments needed in PTM service delivery. The PI determined that for purposes of introducing change within her health network it was not necessary to focus on engaging a broader set of stakeholders with the findings; her authority permitted direct action on these changes [‘It is within my direct reach, because I am the one who says what needs to be done (in my health network)’—PI]. The PI reported implementing several administrative and service delivery adjustments within her health network through ‘micro actions’, as the findings emerged—this was the only case in which ‘real time’ changes were made. While the findings documented in the final report identified service delivery gaps in other health networks as well as implications for higher level change (e.g. municipal health secretariat, health insurers), the PI mentioned that she never intended to push for remedial measures beyond her network, citing lack of legitimacy in assuming such an oversight role:

*I can do it [introduce a change] in my health network, I can do it where I am a manager. However, data about other institutions [e.g. other health networks, the health insurance companies] were generated, [but] I don’t have influence there* (principal investigator).

While the PI notes that the issue of cervical cancer is generally perceived as low-priority within the municipality, this appears to have been a top priority programme for the PI as the findings motivated her to implement decisive action within her network: changing hours of operation to accommodate service users’ needs; negotiating arrangements with health insurers to shorten delays in accessing services for patients and simplifying registration and billing processes to reduce wait times (See Box 3 for detailed descriptions of EIR features).

**Box 3 Detailed description of EIR features in Colombia Case**

**Role of decision-makers**

The DM PI assumed a highly engaged leadership role in this endeavour, driven in part by her strong interest in the PTM programme, which she helped launch. While she was not directly involved in all aspects of the research (e.g. data collection), she reported having led the research activities while remaining abreast of emerging results and assuming responsibility to act on findings. As remedial measures were focused within the DM’s purview—i.e. within the health network over which she has direct authority—she reports being able to directly decide on adjustments to service delivery, which did not require intensive dissemination efforts or stakeholder consultation to make an adoption decision. Reflecting on her experience, she mentioned the importance of sound (ethical) judgment by DM investigators in the use of findings, as well as the capacity to acknowledge service weaknesses.

**Collaborative research partnership**

The DM formed a solid partnership for this study, establishing clear objectives and division of roles/responsibilities. Both co-investigators reported having contracted a strong support team (logistics, methodological expertise), and establishing a clear programme of work. While the co-PI was not a career researcher, his background in epidemiology and decades of research and programme experience appear to have allowed him to serve as a strategic advisor with insider perspective and as a well-respected interviewer. As with other teams, co-PIs did not report having faced significant conflict or other obstacles to collaboration.

**Positioning of research within programme**

While there is no indication that the EIR was deliberately integrated into ongoing programme processes, the fact that it was conducted in a setting with strong quality improvement and performance monitoring processes likely facilitated this research endeavour. Such an environment that values data to improve performance, likely helped foster receptivity of frontline staff to the overall improvement endeavour.

**Implementation Focus**

The original research interest was epidemiologically oriented (case–control study), focusing on measuring coverage of cervical cancer screening and identifying factors associated with low utilization. With TA the research was reframed into a qualitative study focused on the effect of access to and quality of services on coverage. Characteristics of the new study topic appear to have influenced the PI’s ability to launch change processes: programme implementation was highly bounded (i.e. offered within one of five municipal health networks) and the study focused narrowly on identifying problems of access and quality, limited to one programme component (screening). To respond to the implementation gaps uncovered by the study, the co-investigators highlighted the need to acknowledge and confront these service ‘failures’.

#### Case 3: DR male contraception

The DR team conducted a mixed-methods (Qual → Quant) study to identify facilitators or barriers to the effective integration of men as beneficiaries in the national Family Planning Program (FPP), with a specific focus on male contraception. When the PI conceived of the research idea, she was a high-level decision-maker within the Sexual and Reproductive Health programme (responsible for FPP). She initiated the research partnership, inviting two gender studies academic investigators to join as co-investigators. Following her reassignment to the Ministry of Public Health (MoPH) Office of Gender and Development, the PI invited the new SRH Coordinator to join as co-PI, recognizing the importance of her involvement in protocol development and ensuring use of findings. Interviews with the research team members indicate that all four investigators engaged actively in the research and post-research phases. The documented findings focused on the limited availability of male contraception, service delivery factors that impede access to FPP by men and emerging demand for contraception services among young men. Overall, findings were perceived by all co-investigators and most external stakeholders as confirming previously tacit knowledge about the lack of gender lens in FPP and as accurately capturing their lived reality; ‘it is one of the few studies we have that reflect the reality of what is happening with family planning for men’ (Ministry decision-maker). This endeavour was observed to have raised attention to the issue of male contraception and shifted stakeholders’ understanding of the research issue:

*This research has helped me a lot in refocusing what the [FP] program is and in emphasizing that population [men] and that need, the niche that exists and that need—that they [men] want it [contraceptives]* (FPP decision-maker).

Two salient contextual factors reported by various respondents that appear to have complicated this effort are the creation of a new health system governance structure mandated to oversee service delivery^3^ and the strong socio-cultural barriers rooted in conservatism.

Following necessary ministerial approvals, the team organized a formal dissemination meeting—attended by the health minister and other high-level decision-makers—and subsequently convened a multi-sector problem-solving workshop to address study findings. Though the co-investigators reported that the resulting joint action plan proved difficult to implement, the decision-maker co-PI described how she helped advance incremental steps towards introducing changes to FPP. She incorporated key findings in drafting new normative guidance that addresses male contraception (FP service delivery protocol and health provider consultation guide) and also ensured study findings were considered in development of the MoPH 2019 annual plan—this resulted in allocation of resources for activities to build system readiness for change (See Box 4 for detailed descriptions of EIR features).

**Box 4 Detailed description of EIR features in DR Case**

**Role of decision-makers**

Two health system DMs participated as co-PIs on this team. Following reassignment to the Gender Office, the DM PI maintained this role in the team and invited the new SRH coordinator as co-PI, fostering ownership over the findings at the locus of change (i.e. FPP). Both DM co-PIs were observed to have collaborated in strategizing for change from within and outside the programme and remained actively engaged in the post-research efforts. Though the FPP DM comments suggest she was not immersed in all aspects of the research, there is evidence that she served as an integral partner, providing guidance throughout the process with her in-depth understanding of the feasibility of different actions. The FPP DM revealed that her own understanding of the problem shifted in light of the study findings, motivating her to take initial remedial measures.

**Collaborative research partnership**

This team reported having established a strong, productive research partnership between academic investigators and health system decision-makers. This partnership was based on long-standing pre-existing relationships, which facilitated tight-knit, trust-based team dynamics (a ‘sisterhood’ according to the DM PI). Some stakeholders expressed some apprehension about the lack of gender balance within a team studying gender inclusivity. While the DM PI assumed formal responsibility for the study, team members settled into a leadership dynamic described as ‘horizontal’—i.e. less concentrated within the DM PI and distributed across members. This appears to have enabled all team members to pursue distinct, yet complementary strategies to influence change, engaging different sets of stakeholders through their professional networks. The researcher co-PIs self-identified as ‘applied researchers’ and saw it natural to continue their active involvement in post-research problem-solving. The importance of frequent informal communication and linkages among team members, the complementarity of roles and capacities, the personal commitment to the issue based on a shared feminist perspective, as well as the iPIER TA support were described by co-investigators as central to their strong partnership.

**Positioning of research within programme**

This research endeavour did not explicitly seek to integrate research activities into ongoing programme processes. However, its use of interviews with major decision-makers appears to have contributed to positioning these critical implementation stakeholders inside the investigation, heightening their awareness of its aims and processes. Linkages with programme decision-making processes occurred through the FPP DM, who describes her efforts to ensure study findings were considered in annual planning activities.

**Implementation focus**

This case illustrates a less common approach to IR, focusing not on implementation of an ongoing programme, but rather on how to integrate a new service component (male contraception) more widely in the public health system through the FPP. The initial interest was to study perceptions about access to contraception across all potential beneficiaries; with TA support, the team reframed the research question on identifying the programme mechanisms that could incorporate men as direct beneficiaries of FPP, by exploring programme decision-maker and service provider perspectives, in addition to those of male beneficiaries. This refocusing was understood by DM co-investigators as emphasizing an inward view on the programme itself, requiring DMs to acknowledge deficiencies in the ‘supply’ of health services. Many respondents mentioned that the resulting evidence confirmed prior tacit knowledge about a problematic lack of gender focus within the FPP.

### Cross-case analysis

The three cases were markedly different, not only in terms of the research topics, EIR team composition and health system contexts, but also in terms of how EIR features were operationalized. Both ‘most likely cases’ (Colombia, DR) made significant advances regarding research use for decision-making; while the ‘least likely’ case (Bolivia) was able to stimulate one-off reactions to the study at the local level, there was a notable absence of more systemic or long-term change. In this section, we highlight the dimensions of each feature that appear to bear most prominently on the EIR processes that may have contributed to this.

#### Role of decision-makers

Benefits to the research process arising from decision-maker involvement were observed in all cases, though these varied depending on a few conditions. All PIs actively led the endeavour throughout research and post-research phases. However, the positioning and authority of the decision-maker in relation to the programme differed: in Colombia and DR, PIs held some degree of direct authority over the programmes studied; in Bolivia, the position of PI as a provider at a first-level hospital (i.e. above the level of intended programme implementation) meant she lacked the authority to influence wider decisions about implementation. In all cases, the decision-makers were observed to have ample professional networks, which they leveraged to engage key stakeholders at various stages of the research and research use phases. Furthermore, decision-makers in all cases cited the importance of commitment and openness to uncover implementation weaknesses as a factor underpinning effective decision-maker involvement in EIR. Lastly, these cases demonstrate how the decision-maker’s position in the system can also set limits to the roles they can assume legitimately to influence change; e.g. inability to spur action beyond their decision-making purview (Colombia) or to convene key decision-makers in problem-solving based on the evidence (Bolivia).

Our cases highlight key facets of ‘meaningful’ engagement by decision-makers: (1) interest in obtaining pragmatic evidence to guide problem-solving and the associated sense of ownership over the endeavour (2) continuity of engagement throughout research and post-research stages, adjusting intensity of involvement as needed (3) ability to provide intellectual leadership and direction.

#### Collaborative research partnership

All partnerships were initiated by decision-makers specifically for the purpose of this research endeavour; however, only one case directly engaged both career researchers^4^ and decision-makers as co-PIs (DR). In other cases, methodological expertise was filled by short-term contracted technical staff or the iPIER TA providers. Nonetheless, these partnerships brought together stakeholders with different backgrounds and system perspectives who may not otherwise engage in joint problem-solving.

The most prominent elements of the collaborative partnerships across cases relate to commitment, clear and shared purpose and representation of diverse perspectives. The ‘opt-in’ nature of these partnerships, where both PI and co-investigators perceive incentives to participate, appears to play an important role in creating a sense of commitment to the wider team (Colombia, DR). In addition to motivations stemming from the prestige of a WHO/PAHO grant, respondents reported key incentives including technical interest in the issue; personal conviction about the need to act and ideological motivations. From the outset, the purpose of partnership formation was clear: to generate research to improve a specific programme/policy issue identified by the decision-maker. All teams incorporated members with diverse perspectives, from different levels of the system, different government institutions and varied professional linkages across different sectors (e.g. media, industry, non-governmental). The partnership arrangements, however, differed considerably. For instance, in Colombia the PI assumed primary responsibility for key decisions whereas the DR team established a more distributed leadership across co-PIs. Furthermore, while these teams may have experienced some of the commonly cited barriers to collaboration (e.g. time burden; competing priorities; staff turn-over; etc.), they were not reported as impediments to collaboration.

#### Positioning of research within programme (integration)

In our case studies, deliberate ‘integration’ of research and programme processes was generally weak or piecemeal. In DR, some degree of integration was observed when evidence dissemination aligned with established decision-making spaces (e.g. annual planning and budgeting processes or technical working group meetings); this was primarily the result of a proactive facilitating role by decision-makers. The Colombia study, conducted in a context with strong quality improvement processes, reveals potential value in harmonizing EIR processes with ongoing QI cycles, when these are already well-established and supported by adequate information systems.

#### Implementation focus

Most teams grappled with the process of reframing their research questions to ensure clear articulation of ‘implementation’ issues. At times, this required a deliberate shift away from more familiar epidemiological research paradigms (Colombia). All three IR studies focused on identifying problems with ongoing implementation. This demands that decision-makers are able to ‘look internally’ (PI respondent) at service or policy implementation with a ‘self-critical’ lens (co-investigator respondent) so that they are willing to acknowledge implementation weaknesses. Homing in on implementation issues required insider knowledge of programme realities and capacity to use available information in detecting service delivery problems amenable to research. This demanded proactive engagement and direction by decision-makers as knowledge users.

## Discussion

These case studies show how the four proposed EIR design features were put into practice, providing a more nuanced understanding of how this EIR model works in different settings. We discuss insights about the relation between EIR features and research use processes and programme improvement outcomes and also highlight prominent enabling conditions for each.

### Influence of EIR features in evidence-to-action processes

#### Role of programme/policy decision-makers

The role for knowledge users (in our study, decision-makers) in research partnerships both during and after the research emerged as the critical feature driving evidence-to-action processes, as other studies have also shown (Lomas, 2000; Boaz et al., 2015 ; Heaton *et al*., 2015). Other collaborative research approaches also seek to engage decision-makers alongside researchers (Gagliardi *et al*., 2015); the EIR model studied here differs in that it places decision-makers in a lead role throughout the research cycle, as opposed to more intermittent or partial involvement. While the benefits of decision-maker involvement in research have been discussed in other studies (Ross *et al*., 2003; Mitchell *et al*., 2009; Hofmeyer *et al*., 2012; Kok *et al*., 2016 ; Williamson *et al*., 2019), our cases suggest potential added-value of ‘decision-maker-led’ partnerships which promote intellectual leadership and ownership over the endeavour by programme/policy actors that are in a position to apply the evidence.

In our study, the effect of decision-maker involvement on research use spanned several dimensions. Decision-makers identified and pursued the research topic with the explicit aim of improving their programme, ensuring the research was responsive to their information needs—a determinant of research use frequently cited by decision-makers (Oliver *et al*., 2014; Kok *et al*., 2016 ; Williamson *et al*., 2019)—and also instilling a sense of ownership over the research. Decision-makers in a lead role directly accessed the evidence (as it emerged) and could reflect on its implications for the programme/policy given the local context (Morton, 2015), often shifting their understanding of the problem or solution. Decision-makers often express frustration that research fails to provide clear or relevant recommendations for action (Williamson *et al*., 2019); placing decision-makers in a central role instead empower them as change agents to devise feasible solutions based on the evidence.

The breadth of the decision-maker’s professional networks and their ability to interact with other potential knowledge users are key in influencing research use *beyond the PI’s sphere of influence*. Well-connected decision-makers leveraged their professional networks, influencing other pivotal implementation stakeholders to engage with the evidence in problem-solving outside decision-makers’ sphere of authority. The importance of decision-maker ability to engage others in using the evidence is also reported in other studies (Bowen and Zwi, 2005; Hinchcliff *et al*., 2014; Kok *et al*., 2016). However, as demonstrated in the case of Bolivia, while decision-maker professional connectedness may benefit dissemination of evidence by engaging diverse stakeholders, at times this is insufficient to compel stakeholders to action.

Decision-maker PIs adequately positioned and supported to act on the evidence directly in their own work, holding the authority/influence to make critical programme decisions and mobilize resources ( Kok *et al*., 2016), are more likely able to catalyse programme improvement processes. In some cases, this may involve taking action as the evidence emerges in ‘real time’. The capacity of the decision-maker to proactively identify windows of opportunity for action in broader decision spaces or processes is also key. These findings echo (and confirm) the need for careful selection of the decision-maker PIs for EIR initiatives, as suggested by others (Haynes *et al*., 2018). Key enabling conditions for decision-maker involvement were identified through our case studies: decision-maker authority/influence over the programme; decision-maker commitment based on a pragmatic need to resolve a problem and critical reflection to acknowledge implementation weaknesses.

Specific aspects of the iPIER grant mechanism may bear upon this interpretation. The decision-makers made a deliberate choice to assume a central role in the research (i.e. voluntary participation); furthermore, the fact that the research topics were identified by and of genuine professional interest to the implementers (rather than imposed) suggest the importance of self-selection in the quality of their engagement.

#### Collaborative research partnership

The collaborative research partnership is one approach to promoting ‘interaction’ between researchers and decision-makers which the literature shows is central to evidence-informed decision-making (Lomas, 2000; Denis and Lomas, 2003; Ross *et al*., 2003; Mitchell *et al*., 2009; Oliver *et al*., 2014; Haynes *et al*., 2018). Various forms of multi-stakeholder research partnerships have also demonstrated beneficial effects in evidence-to-action endeavours (Hinchcliff *et al*., 2014).

In our cases, the effects of these research partnerships on research use centred around the ‘conceptual’ dimension of ‘impact/use’ (Weiss, 1979; Nutley *et al*., 2007), generating new understanding or ways of thinking about the issue among key stakeholders. Research teams promoted the use of evidence by harnessing the diversity in team members’ perspectives or cultivating external stakeholder relationships and networks.

Teams able to maintain a focus on a common purpose fostered productive interactions among members despite differing viewpoints, in some circumstances contributing to pursuit of multiple complementary strategies to promote evidence use. Furthermore, the ability to leverage different co-investigator professional networks facilitated strategic engagement of diverse system stakeholders in research linkage and exchange efforts (e.g. multi-sector stakeholder consultation workshop in DR). This echoes studies that have shown increased likelihood of research use when obtained from trusted ‘interpersonal channels’ (Haynes *et al*., 2018), and suggests professional connectedness (reflecting respect and trust) is an important consideration in selecting co-investigators. Our cases have shown the benefits of the collaborative nature of EIR in stimulating the ‘social life of research’ (Haynes *et al*., 2018), whereby new ideas arising from the evidence are circulated, raising attention to and fostering discussion about critical findings—which can help catalyse significant programme change.

The linkages between the collaborative research partnership feature and programme improvements observed in our case studies appeared to stem primarily from the decision-maker PI role in the partnership. In two cases (DR, Colombia), the effects of decision-maker leadership on the success of the partnership was clear. Strong leadership and direction by the decision-maker brought clarity of purpose to the research endeavour (to support the PI in their decision-making), prioritizing their information needs. This strong orientation towards decision-maker needs may have precluded major disagreement or extensive debate that might arise in a more ‘egalitarian’ partnership where multiple priorities/perspectives compete (Sibbald *et al*., 2014; Heaton *et al*., 2015).

However, perceptions about the interests represented within the study team can also influence how the research is received (and used or not used) by key system stakeholders (Morton, 2015). In the case of DR, the absence of men on the study team appears to have raised some apprehension among some stakeholders concerning the legitimacy of the team’s pursuit of a gender lens. In Colombia, participation of co-investigators with different interests (i.e. municipal health office and the programme) appears to have enhanced the credibility of the research among stakeholders—with the participation of a more neutral, respected co-investigator perceived as balancing out perspectives.

It is important to recognize the role of iPIER TA in these projects. In addition to providing methodological support, the TA influenced partnership dynamics, in some instances perhaps facilitating the collaboration. Regular TA check-ins were reported to exert positive pressure on the teams to maintain momentum (e.g. meet deadlines) and foster cohesion. TA likely also contributed to circumventing many challenges with collaborative research reported in the literature (Bowen *et al*., 2016; Rycroft-Malone *et al*., 2016; Nyström *et al*., 2018). In contexts where such external support is absent or where the appropriate skill mix is not represented in the partnership, such challenges would likely require greater effort to address.

#### Positioning of research within programme (integration)

The notion of integrating research into programme/policy processes is critical to its ability to inform decision-making (Walley, 2007; Ghaffar *et al*., 2017). Positioning of research within ongoing programme/policy processes requires deliberate strategizing and coordination and is critical to institutionalization of EIR. While these processes are complex and challenging, advances are being made and documented (Awoonor-Williams and Appiah-Denkyira, 2017; Hirschhorn *et al*., 2017). Evidence of ‘integration’ was limited in our cases, hindering our analysis of its effects on research use and programme improvement. It is possible this notion of integration was not clearly articulated or emphasized in the guidance to grantees as it was not explicitly mentioned by study team respondents when asked to describe EIR. However, the fact that two cases (DR, Colombia) made significant advances in promoting evidence use and catalysing changes to the programme without fully integrating research and programme processes suggests that this may not be an essential feature for EIR. The EIR projects studied were one-off efforts, conducted by novice IR teams, for whom institutionalization of IR was not a priority. Our findings suggest that in such circumstances, full ‘integration’ may not be necessary. Involvement of well-positioned decision-maker PIs—ensuring the research is responsive to problem-solving needs and later incorporated into key decision-making processes—can help align research and programme processes, as others have also described (Desimone *et al*., 2016).

#### Implementation focus

A research focus on implementation issues lies at the foundation of IR and is pivotal for identifying programme improvements that can lead to strengthened system performance and better health outcomes (Ghaffar *et al*., 2017; Theobald *et al*., 2018). By definition, an implementation focus considers the nature of the programme, processes through which they are delivered, the role and responsibility of actors at different levels, and the context in which this occurs (Damschroder *et al*., 2009). Others have shown that an implementation focus results in substantively different research questions as compared with other approaches to programme improvement (Rao *et al*., 2016). Careful formulation of the research question(s) is needed to ensure results are relevant and actionable within the local reality for programme improvement.

In our case studies, the implementation focus was explicitly intended to support decision-makers to improve ongoing programme implementation and directly influenced EIR processes at multiple levels. Such an orientation helped: (1) identify specific barriers to or deficiencies in programme/policy delivery, revealing concrete opportunities for direct action by decision-makers and (2) highlight the contextual factors that bear upon implementation. Decision-maker leadership in identifying research questions, discussed previously, is key. The novice EIR teams in our study demonstrated challenges in achieving an implementation focus, even in the context of tailored TA; this suggests that without such guidance other teams new to IR may struggle to grasp this research orientation.

In our cases, the need for data about implementation realities influenced the research conduct, e.g. by engaging system stakeholders involved in implementation or programme/policy decision-making as interview respondents. Such participation appears to cultivate their buy-in vis-à-vis findings, in some cases influencing their understanding about the implementation problem (DR, Colombia) and even ‘awakening key actors to action’ (PI respondent). Furthermore, interviews with stakeholders can make tacit knowledge about implementation explicit, helping document and formalize ‘lived experiences’ concerning implementation that can then be considered in decision-making processes. Such interviews may also elicit recommendations for action from implementation stakeholders—constituting a one-on-one consultative problem-solving exercise that can generate immediately actionable findings for programme improvement.

### Further research

Our findings suggest that in addition to the EIR features studied here, there are other factors that influence EIR, such as those related to the local context and to the ability of EIR teams to identify and act on opportunities to apply their research in programme/policy decision-making. To understand more fully how EIR ‘works’, further case study research is needed on the strategies EIR teams use to leverage local conditions, resources and opportunities in their pursuit of evidence-informed programme improvement.

### Implications

Table 3 summarizes key lessons gleaned from our research for the design and implementation of EIR, providing guidance for the application of EIR features and for creating an appropriate enabling environment. 

**Table 3:** Considerations for application of EIR

**Implications for EIR practitioners**
*Decision-maker role* The degree of influence/authority of decision-maker PIs over the targeted programme/policy plays an important role in the success of the research in improving programmes Intellectual stewardship and direction by decision-maker PIs throughout the research and post-research processes has shown substantial benefits to the EIR endeavour The nature of decision-maker PI involvement in the research varies by context (e.g. varying intensity of engagement by stage of the process) and may depend on ethical considerations such as generating bias through participation in data collection
*Collaborative research partnerships* EIR requires research teams with appropriate mix of skills, technical expertise and professional perspectives/networks, relevant for both research and post-research phases Strong partnerships build on existing relationships of trust and respect In selection of co-investigators, the strategy of ‘opting in’ to the research project may help ensure committed team members While partnership arrangements for EIR may vary across projects, they should ensure role clarity and establish shared objectives/expectations among team members
*Positioning of research within programmes* Formal integration of research and programme processes may not be essential for all EIR projects, particularly where the decision-maker PI assumes a role that ensures alignment of the research with programme needs It is helpful for EIR teams to consider, at a minimum, timing/cycles of existing decision-making processes or problem-solving mechanisms as they plan their research
*Implementation focus* Understanding how to operationalize a research focus on implementation may require significant effort and support for teams new to EIR EIR that engages system stakeholders as key informants can not only lead to better understanding of implementation issues, but can also raise stakeholders’ attention to the issue and engage them in critical reflection to inform problem-solving EIR teams can identify implementation problems through a range of information sources, such as routine monitoring data, other research/special studies, or experiential knowledge of programme/policy stakeholders EIR requires co-investigators to be able and willing to assume an inward-looking, critical view on service delivery and openness to acknowledge implementation gaps/deficiencies
**Implications for health research donors**	
*Decision-maker role* Calls for proposals for EIR grants should be appropriately channeled to suitable health system decision-maker cadres within ministries of health In establishing selection criteria for proposals to be funded consider the role of decision-maker PI within the targeted programme/policy and ability to act on study findings	
*Collaborative research partnerships* Consider inclusion of co-PIs affiliated with academic or research institutions as a partnership requirement, particularly those with experience in health systems/services research, IR or other applied research	
*Positioning of research within programmes* Formal integration of research and programme processes does not appear to be fundamental for one-off research grants that do not aim to institutionalize EIR	
*Implementation focus* Teams new to EIR require capacity building and technical orientation on the rationale for, purpose, and methodologies appropriate for IR To prioritize demand-driven research with sufficient support, grant mechanisms should encourage applicants to consult with a range of authorities/stakeholders to ensure research projects align with local health research priorities	
*Cross-cutting* Funding is needed to continue support for EIR initiatives in low-resource contexts Systematic evaluation of these endeavours should be built into the grant programme from the outset to continue building the knowledge base about EIR	

### Study strengths and limitations

#### Strengths

This study has several strengths. A data collection protocol guided by a conceptual framework to systematically examine underlying constructs. Comparison of cases in three distinct settings enriched the analytical process. Exploration of ‘researcher’ or ‘decision-maker’ perspectives, as well as other system stakeholders allowed corroboration of evidence. Iterative, prospective data collection over almost three years allowed for prolonged engagement with cases; this strengthened rapport with respondents, permitted follow up on key points after intermittent analytical work. Periodic debriefing with senior researchers helped guide study design, analysis and reporting.

#### Limitations

This study relies heavily on self-reported data by those directly engaged in or implicated by this research experience. Eliciting multiple respondent perspectives helped mitigate this, as did review of documentary evidence. Recall bias was reduced, but not entirely avoidable as it was not always possible to align data collection with the real time experience. The study likely influenced the actions and behaviour of EIR teams, perhaps motivating additional effort after grant completion. The lengthy nature of the change processes involved did not permit analysis of outcomes at health service or system level. The EIR experiences studied do not reflect routine conditions of implementation, given the iPIER grant mechanism and related TA. Key aspects of EIR pose challenges to its study: based primarily on social processes, driven by collective experience and interaction among multiple actors; not always directly observable through tangible evidence; highly complex, context-sensitive; all of which limited our analysis to identifying the contribution (rather than attribution) of EIR to the effects observed. We opted to examine three of the seven 2016–17 EIR projects with the aim of greater analytical depth; this limits the transferability of our findings to the entire cohort.

## Conclusion

This comparative case study analysis contributes to an emergent body of knowledge about how EIR influences evidence-to-action processes. We examined four EIR features—central involvement of decision-makers in research; collaborative research partnerships; positioning of research within programmes and research focus on implementation—to draw conclusions about how EIR affects research use and policy or programme improvement. Distinguishing itself from other work that promotes the embedding of researchers into programmes (Denis and Lomas, 2003; Vindrola-Padros *et al*., 2017; Wolfenden *et al*., 2017) or other modes of engaging knowledge users in research (Gagliardi *et al*., 2015), our study indicates that when well-supported, decision-maker-led IR partnerships can stimulate the use of research to drive programme improvements.

This study expands upon previous analyses of EIR (Langlois *et al*., 2019) to reveal how the EIR features were operationalized and contributed to demand-driven, action-oriented research that could be applied by both study teams and other system stakeholders. Our findings highlight the importance of a lead role for decision-makers in EIR, supported by decision-maker authority over the programme studied, professional networks and critical reflection. Strong research–practice partnerships were facilitated by a clear and shared purpose, representation of diverse perspectives and commitment. The weak evidence about positioning research within programme processes, suggests this feature may only be relevant in specific circumstances; decision-makers can contribute to this integration. Implementation focus—fundamental to EIR—demands proactive engagement by decision-maker PIs in conceptualizing the research and identifying opportunities for direct action by decision-makers.

As the EIR approach is far from ‘business as usual’ in low-resource contexts, key supports are needed in terms of both capacity building for health sector stakeholders involved as well as system-level strategies to create an enabling environment—we have highlighted key considerations intended to advance the practice of decision-maker-led EIR.

## Supplementary Material

czaa126_Supplementary_Data

## Footnotes

1 Argentina, Bolivia, Brazil, Chile, Colombia, DR and Peru.

2 Decision-maker leadership in initiation of response to iPIER call for proposals, conceptualization of research topic/questions, self-designation as PI, positioning within targeted programme/policy; and stage reached at grant completion.

3 The National Health Services unit.

4 Those affiliated with academic or other research institutions, pursuing a career primarily focused on the design and conduct of research.

## Notes

*Ethical approval.* A non-human subjects research exemption was received for this study from both the PAHO Ethical Review Board and the Johns Hopkins School of Public Health Institutional Review Board.
