# Supplementary material for: How does embedded implementation research work? Examining core features through qualitative case studies in Latin America and the Caribbean
Source: Health Policy Plan. 2020 Nov 6;35(Suppl 2):ii98–ii111. doi: 10.1093/heapol/czaa126 (PMC7646734; doi:10.1093/heapol/czaa126)
Supplement: czaa126_Supplementary_Data [file czaa126_supplementary_data.zip › czaa126-suppl_data/Supplement3.docx]

**Supplement 3: Illustrative Semi-structured Key Informant Interview Guides**

*Respondents: Study Team Members (Decision maker Co-PI and researchers, with adaptions]*

**Interview 1: Situating each team and advances since grant completion**

Interview Topics

1. Update on stage of process

- Could you please share with me an update on how things have progressed (or stalled) since the iPIER project formally ended in Sept/Oct 2017? What key events or activities followed after the development of the final iPIER report with findings and recommendations (if unclear, probe on when the final report was completed)?
  - Probe whether they have conducted any dissemination activities (meetings, workshops), developed any knowledge/communication products, begun processes to apply changes to program, etc.]
  - Check for any changes in the role/position of the respondent and how this has affected their involvement in the activities that followed; check whether both researcher and decision maker Co-PIs are still engaged in relevant activities (i.e. whether the ‘collaboration’ is still active—if so, probe to understand how they have managed to continue to keep the collaboration alive and in what ways the collaboration dynamic has shifted).
    - If team has disbanded (or members no longer engage regularly), probe regarding the factors that led to the dissolution of the team and abandonment of activities.
  - Probe to ascertain the key advances/achievements made since the research project formally ended [*if needed, clarify that ‘advances’ are with respect to the application/use of the evidence for improving the program*].
  - Probe to ascertain any important obstacles or challenges confronted and whether/how they were overcome.
  - Probe: When the grant ended, was a decision made among the team members regarding next steps and how to carry out subsequent stages of work to ensure the evidence/ recommendations were used to improve the program? [Determine whether an action plan or some other strategy was developed—include in doc review if so.
  - Probe to understand what specific activities are being pursued presently and how much time they are able to dedicate to this work, given other responsibilities?

1. Background on the selection of research topic

- [Program and PH Agenda] Thinking about X program within the broader health sector context of X municipality/province/etc., how would you rank its level of priority on the local public health agenda? What about on the national health agenda?
  - Note whether this this changed at all over the course of the project, if so, why.
  - Probe: How would you describe the level of public attention this program receives in terms of its performance, service delivery, or other quality issues in recent years?
  - Probe: Are there any other formal, ongoing efforts focused on improving some aspect of this program?
- [Problem Identification—mainly for Decision Maker respondents] Going back to the beginning of this project, when you were identifying the research topic and questions for your program, what would you say were the key driving factors for selecting the specific topic you chose (e.g. previous research evidence; anecdotal knowledge; policy changes; etc.)?
  - Did the focus of the research change significantly after the protocol workshop and discussions with INSP? If so, how was this change received among broader circle of program stakeholders?

3) Follow up on Stage 1

- [RE: Process of co-production of research]
  - Considering the collaborative nature of this research team (decision-makers alongside researchers), what kinds of factors or circumstances do you think were most helpful in ensuring that the team was able to complete the research and produce preliminary recommendations?
  - What was most challenging or frustrating about this experience of conducting program research in collaboration with decision makers and researchers? If you were to repeat this, how would you do things differently to minimize/avoid these challenges?
- [RE: Intermediate outcomes: Evidence] Thinking back to the findings that were produced through the IR, how useful do you think these have been (will be) in guiding improvements to X program? How relevant are they to the program problem initially identified?
  - Probe: Do the findings target aspects of the program that can actually be changed/acted upon? I.e. is the evidence in reference to aspects of the program that fall under the purview of those involved in the research (or are does the evidence point to higher level issues that require higher level stakeholders, more complex decision making processes)
  - Probe on interpretability of findings by key stakeholders; volume and complexity of evidence presented.
  - Probe: How credible do you think other key program stakeholders (not involved in the research—e.g. other program decision makers, politicians, the public, etc.) have found this evidence? Have you observed any issues around the credibility of the evidence?
  - Explore whether any findings were unexpected /surprising or contrary to other knowledge about the program.
- [RE Processes: Collaborative approach] Do you think the fact of having collaborated with researchers/decision makers in this research project has had any influence (either positive or negative) on how subsequent activities (e.g. dissemination, problem-solving, stakeholder consultations) have been planned or carried out? If yes, can you share specific examples/details that illustrate this?
- Based on your experience, what advice would you give a new iPIER grantee on how to ensure successful collaboration between researchers and decision makers in conducting IR for program improvement?

**Interview 2: Engagement with research**

- [Intro to be tailored based on findings from Interview 1]
  - First check whether any new advances have been made since Interview 1. If none mentioned, or not clear, ask: Have any activities been implemented to make sense of the evidence produced through iPIER or to plan/strategize how this evidence can be put to use [e.g. reviewing findings, weighing the evidence, problem-solving, developing recommendations, etc.]?
    - Ascertain respondent’s involvement/role in any activities /events mentioned (e.g. presentation of findings to stakeholders; problem solving or negotiation activities aimed at devising improvements to program; etc.).
    - How effective do you think X activity [or the broader dissemination strategy, as appropriate] has been in advancing the use of the IR evidence produced to improve X program?
    - [In the case of low effectiveness:] At this stage, if you were going to enhance the use of the evidence produced through iPIER, what would need to happen?
- Shifting our glance to the original iPIER research team, how would you describe the interaction among the iPIER research team members following the close of the grant—what happened to the team?

**Probes:**

- - [Assuming some form of continued engagement among team members:] How would you compare the current dynamic between team members to the dynamic that existed during the research phase? What has changed, what has continued? Probe to understand the mechanisms or communication strategy that continues to link researchers and decision makers.
  - Probe: Who is directing/leading the effort at this time? What does X’s leadership role entail at this stage [ask them to mention specific tasks/responsibilities: eg. planning and organizing activities, liaising with key stakeholders, etc.]?
  - Probe whether the relationships established or experiences shared during the research collaboration have played any role (positive or negative) in the current stage of the process.
- Have other program stakeholders (or other decision makers) been engaged in the activities following the production of findings?
  - Probe: If so, who are some of the other stakeholders that have been (or are expected to be) involved in responding to the research findings? How did these stakeholders become engaged (who initiated their engagement)?
  - Probe: In what ways [through what mechanisms] have these external stakeholders been able to engage with the evidence—what do you see have been the most effective strategies of engaging them? How has the involvement/contribution of such program stakeholders impacted the way the IR evidence is perceived or applied (can you share an example)?
  - If no other stakeholders have been engaged, probe to understand if this was deliberate, and if so, what the motivations were behind this approach. If not deliberate, what kinds of challenges/barriers did they face in engaging other key stakeholders?
- Through these activities [aimed at communicating or consulting about the evidence], does it appear that the IR evidence has had an effect on how key program stakeholders (internal and external) understand the program and/or think about potential solutions or viable program improvements?
  - What kinds of changes have you observed among the most critical decision makers?
  - What would you say are the key factors/circumstances that enabled key stakeholders to engage with the evidence and devise potential solutions [NOTE: this is not about implementing solutions/changes, but about conceptualizing/developing them]
- What kinds of challenges/barriers (either at the individual level or the organizational/program level) have been faced in trying to use the evidence to devise solutions/strategies to improve the program?
  - Probe: How did aspects of the political or social environment help or impede the use of evidence to guide program solutions (problem solving)? (Note: the **political environment** could pertain to the influence of local government officials, election cycles, resource allocation decisions within your program or institution, etc. ; **social environment** could pertain to lobby groups, media coverage, civil society groups, public opinion, etc.)
- What is the vision/plan for moving things forward from here on?
  - Probe: is there a common vision among the team members? Divergent views?
  - What do you expect will be the most important challenge to be faced in the next phase?
- How would you describe what ‘success’ of the iPIER initiative would look like for X program? Please be as specific as possible in describing what it would entail.

**Interview 3:** Actions/Changes to program or decision making actors/processes & final thoughts

*[Additional questions will be formulated at a later stage of the research in order to tailor questions to the processes and outcomes experienced by each case, as well as to the specific respondent categories. Broadly, the following types of questions will be addressed.]*

- Considering the initial aim of the iPIER project—which was to stimulate the use of evidence to improve health programs—in your opinion, what do you see as the most important outcomes of this process to date? [Consider outcomes related to decisions about program changes; or to thinking/understanding about the program among key stakeholders; or to broader shifts in how stakeholders understand the role of research evidence for program improvement; etc.]
  - Probe: Were any key decisions taken to improve X program? [Probe: On what basis were such decisions made (or through what processes of negotiation)? Do you think the initial researcher-decision maker collaboration influenced these processes in any way —positively or negatively? How?]
  - Probe: Over the course of this endeavor, have the opinions of key decision making stakeholders shifted regarding what is needed to improve this program (i.e. the most appropriate strategy/changes needed to improve the program)? How? Has your own personal stance shifted (how)?
- Considering the typical approaches to program review or program evaluation/improvement in your health system, what do you see as the most critical differences (if any) between the effort initiated through iPIER and ‘business as usual’?
  1. Explore whether other collaborative work has emerged from this experience; whether relationships have expanded beyond the work related to iPIER; whether any new mechanisms or structures for use of evidence in decision making have been established, etc.
- [Personal impact—researchers]: Has this experience in any way influenced your views on how to ensure that the evidence you produce, as a researcher, can be used for programmatic improvements?
- Based on your knowledge of how things typically work, what might have been the mostly likely course of events for the program in the absence of this evidence about program implementation (e.g. status quo; changes made based on other available information; pursuit of funding elsewhere to conduct research, etc.)?
- [Personal impact--decision makers]: Has this experience in any way influenced your views on the role for evidence in program improvement? How about on your thoughts on effective strategies to incorporate research into program review efforts?
- If you were asked by the Minister of Health to replicate this IR effort for program improvement elsewhere, what would you do the same and what would you do differently?
- Are there any other comments you’d like to share about this research uptake effort and how it was implemented in your context?
